# Mr. Clutterbuck, on Tetanus

**Published:** 1800-10

**Authors:** H. Clutterbuck

**Affiliations:** Walbrook


### To Dr. BRADLEY.

Dear Sir,

THE following Cafe, which, during a confiderable part of its progrefs paffed under your notice at the Univerfal Difpenfary, will not, I flatter myfelf, be deemed unworthy a place in the Medical and Phyfical Journal. It exemplifies, I think, the good effects of two powerful articles of the Materia Medica, *cold affusion* and *lead*, in difeafes of the fpafmodic clafs.

I am, your's, with refpect,

*Walbrook, Aug.* 15, 1800. H. CLUTTERBUCK.

---

Sarah Jones, aged ten years, refiding in Ray-ftreet, Cold-bath-fields, was attacked with fymptoms of Cholera in July, 1794; on the following evening fhe complained of univerfal pain of the limbs and trunk, and the next day began to be affected with fpafms in various parts, occurring at uncertain intervals: The fymptoms of Cholera now difappeared. Various antifpafmodic remedies were employed for the fpace of a fortnight, but without any permanent advantage. She had taken opium in large and repeated dofes, both by the mouth and in clyfter; wine and other ftimulants had been adminiftered in confiderable quantities; blifters had been applied freely; and mercurial ointment had been rubbed into the back and neck in fuch quantity, as to produce forenefs of the mouth. On the 1ft of Auguft the fymptoms were as follows.

I found her fitting up in bed with her head ftrongly bent back on her fhoulders, in which ftate it had continued without intermiffion for the laft two days; her countenance was expreffive of great diftrefs; every five or fix minutes the fpafms increafed in violence, occafioning exceffive pain, and forcing her to fcream aloud; and once in four or five hours a general convulfion came on, in which her body was fo forcibly incurvated backwards, as to caufe it to reft almoft entirely on the head and heels *(opisthotonos)*. This ufually lafted about half an hour, during which fhe retained her fenfes perfectly, crying out with the violence of the pain fhe endured. She defcribed the fpafms as beginning at the pit of the ftomach, and fpreading thence, around the fides, to the mufcles of the back and neck. She complained of much head-ach, and had had little or no fleep for feveral days. She had no appetite for food. The tongue was flightly foul, and there was not much thirft; the pulfe at the wrift was quick, and remarkably foft; the fkin felt flaccid, and there was confiderable tendency to fweating, though without much increafe of heat. During the laft twelve hours fhe had taken a grain of folid opium every half hour, and her mouth was at this time under the influence of the mercury which had been rubbed in. The opium neither produced any remiffion of the pain or fpafms, nor did it induce any tendency to fleep.

It was now refolved to try the effect of cold affufion; and for this purpofe fhe was taken into the cellar naked, and feveral bafons of the coldeft pump water we could procure were dafhed with force over the back and neck: the effect was furprifing and inftantaneous. From the firft application, the fpafms almoft entirely ceafed. She fmiling, faid, "I am quite eafy now." After a few applications of the cold water, a fhivering came on, and fhe begged to be returned to bed, when a defire for food was expreffed, which fhe took freely, and foon after fell into a found fleep.

On the following morning we found her free from pain; fhe had flept well, and eaten heartily. The mufcles of the neck continued fomewhat rigid, but not fo as to bend the head backwards. In the courfe of the day, there appeared a flight tendency to a return of the fpafms at times; and this was the cafe for feveral days after. The application of the cold water was, therefore, directed to be repeated three times a day; and as its effect was apparently diminifhed by habit, it was rendered ftill colder by the addition of ice, procured from a neighbouring confectioner.

Within the fpace of a week, the mufcles of the back and neck ceafed to be fpafmodically affected: The patient, however, ftill complained of violent pain about the region of the ftomach, fpreading thence to the fides, and recurring at uncertain intervals, generally twice or thrice daily; thefe pains were manifeftly aggravated whenever the cold affufion was repeated. In other refpects, the patient was free from complaint, enjoying her appetite and natural reft.

It was now attempted to relieve the fpafmodic pains at the ftomach, by opium and fimple ftimulants; but thefe never produced the defired effect, though exhibited in large dofes; and they manifeftly increafed the difpofition to fpafm. Camphor, given frequently in dofes of four grains, produced no effect on the pains, but was followed by a general efflorefcence on the fkin. The metallic falts were exhibited by turns with better effect, the fpafmodic pains wearing off gradually under their ufe: it was not, however, till the approach of winter, that they ceafed to recur altogether. It is remarkable, that the patient bore large dofes of the vitriols of copper and of zinc, (as much as two grains of the former, and five of the the latter) without having ficknefs induced; yet, a fingle grain of opium never failed to excite head-ach and naufea. It was generally obferved, that when the metallic falts induced fick-nefs, the pain at the ftomach was the fooner relieved.

After paffing the winter without any of her former complaints, except occafional pains at the ftomach, but which did not materially affect her general health; this fame patient, in July following, became again fpafmodically affected, but with fymptoms varying fomewhat from the preceding attack. The mufcles of the neck and back were at times affected, but in a much lefs degree than formerly. Some or other of the voluntary mufcles were generally in a rigid and contracted ftate; fometimes, however, they were all free from fpafm. The muf-cles fituated on the fore-arms were thofe moft commonly affected, the hands remaining clofely fhut, except the thumbs and fore-fingers, which fhe had always the free ufe of. Sometimes fhe had a perfect ufe of her hands for a fhort time, then again they became fuddenly contracted. When forcibly opened, which occafioned confiderable pain, they remained rigid, but clofed again by flow degrees; marking the fpafmodic ftate, as well of the *extenfor* as of the *flexor* mufcles. Different fets of mufcles were affected at different times. Sometimes the head was drawn awry for an hour or two; or the mouth was violently diftorted. At others, the eye-lids were forcibly fhut. Two or three times in a day, or oftener, fhe became generally convulfed, talking wildly and incoherently at the time. Thefe fits ufually lafted about half an hour.

*Cold affufion* was again made trial of, but without its former good effect; and as it never failed to occafion diftreffing pain at the pit of the ftomach, its ufe was not perfifted in. Opium always feemed to do harm, by increafing the irritability, already in excefs. Calomel was alfo employed, fo far as to occafion forenefs of the mouth; but the fymptoms were manifeftly aggravated by it.

Confidering that the difeafe vifibly confifted in a too great difpofition to contraction in the voluntary mufcles, and that *lead*, in its operation on the fyftem, produces ultimately a re-verfe effect to this; finding, too, that the fpafms were aggravated by mercury, a fubftance which feems to be oppofed, in its mode of action, to lead; and that little advantage was gained by the ufual tonic remedies, it was determined to try the effects of *lead*. A quarter of a grain of the acetite of lead (*saccharum saturni*) was therefore exhibited four times a day, and continued for the fpace of three weeks, with little intermiffion. After it had been ufed for the fpace of four or five days, a confiderable amendment was obfervable. The fit ceafed to return, and and the partial ſpaſmodic contractions were much reduced in violence and frequency. At the end of the period above mentioned, the lead began to manifeſt its uſual deleterious effects on the ſyſtem; pains reſembling rheumatiſm were felt about the ſhoulders and fore-arms, and a weakneſs of the wriſt was apparent; coſtiveneſs, with pain in the bowels, likewiſe took place: On this account the lead was diſcontinued, eſpecially as the returns of the ſpaſms were now exceedingly ſlight and rare.

After a fortnight had elapſed, an increaſe of the ſpaſms took place, and the patient appeared relapſing faſt into her former ſtate. The lead was again therefore had recourſe to, and within a week was followed by the ſame good effects as at firſt, the diſorder being much mitigated in violence, but not leaving her entirely, nor did it do ſo till the return of winter, when the ſymptoms altogether, though gradually, diſappeared.

Although the uſe of the *saccharum saturni* was continued for upwards of two months, it did not now produce any ill effects on the ſyſtem. I had an opportunity of knowing, that the diſorder did not return the following ſummer; nor had any ill conſequence ſucceeded the employment of the lead, which was never given in greater quantity than above ſpecified.—I may add, that I have experienced the ſafety and advantage of this remedy in many other caſes of ſpaſmodic affection.

